# The Diagnostic Value of Signal-to-Cutoff Ratios in Architect and Alinity HIV Screening Assays: A 10-Year Experience in a Pandemic-Affected, Low-Prevalence Setting

**DOI:** 10.3390/v17091179

**Published:** 2025-08-29

**Authors:** İmran Sağlık, Melda Payaslıoğlu, Hatice Ortaç, Hülya Ayma Rüzgar

**Affiliations:** 1 Department of Medical Microbiology, Faculty of Medicine, Bursa Uludag University, Bursa 16120, Turkey; 2 Department of Biostatistics, Institute of Health Sciences, Bursa Uludag University, Bursa 16120, Turkey

**Keywords:** HIV diagnosis, signal-to-cutoff ratio, fourth-generation screening, Architect assay, Alinity assay, COVID-19 pandemic, diagnostic accuracy, late diagnosis

## Abstract

Early and accurate diagnosis of HIV remains a cornerstone of public health strategies. This study aimed to evaluate the predictive value of signal-to-cutoff (S/CO) ratios from two fourth-generation HIV screening assays (Abbott Architect and Alinity) and to analyze diagnostic trends across pre-pandemic, pandemic, and post-pandemic periods in a low-prevalence setting. We retrospectively analyzed 197,642 unique HIV screening tests conducted at Bursa Uludağ University Hospital from 2015 to 2024. Receiver operating characteristic (ROC) analysis was used to determine optimal S/CO thresholds for distinguishing true-positive results. Of the 197,642 samples screened, the overall HIV prevalence was 0.5%, with 196 cases (0.1%) confirmed as new diagnoses. The Architect assay showed an optimal S/CO threshold of ≥11.8 (sensitivity 98.3%, specificity 97.3%). The Alinity assay demonstrated 100% sensitivity and specificity at an S/CO threshold of ≥19.1. Although a temporary decline in test volume occurred in 2020, there was no statistically significant difference in confirmation rates across years. During the pandemic, newly diagnosed individuals were significantly older and had lower CD4 counts, indicating delayed diagnosis (*p* = 0.026 and 0.008, respectively). Men who have sex with men (MSM)-related transmission significantly increased post-pandemic (*p* = 0.032). S/CO ratio–guided interpretation enhances diagnostic accuracy and may reduce unnecessary confirmatory testing, especially in low-prevalence and resource-limited regions. Selecting the optimal threshold can help to ensure a timely diagnosis and optimize HIV screening algorithms.

## 1. Introduction

Human Immunodeficiency Virus (HIV) infection continues to pose a major challenge to global public health [1,2]. According to the World Health Organization (WHO), an estimated 40.8 million (37.0–45.6 million) people were living with HIV worldwide in 2024, with approximately 1.3 million new infections occurring within the same year. The global prevalence among adults aged 15–49 years was estimated at 0.7% (0.6–0.8%) in 2024 [1]. In Türkiye, 1527 individuals were officially diagnosed with HIV in 2024, although the actual number is likely higher due to undiagnosed or asymptomatic cases [3]. In alignment with global goals, WHO and the Joint United Nations Program on HIV/AIDS (UNAIDS) continue to implement strategic plans aimed at ending the HIV epidemic by 2030 [2].

The COVID-19 pandemic further exposed vulnerabilities in HIV diagnostic systems. Restrictions on movement, strained healthcare resources, and reduced clinical availability disrupted routine screening, delaying diagnosis—especially among asymptomatic individuals and key populations. Reports suggest that the apparent decline in new HIV diagnoses observed during the pandemic more likely reflects reduced access to testing rather than a true decrease in transmission [4,5]. These disruptions highlight the importance of diagnostic strategies that are streamlined, adaptable, and resilient during public health emergencies.

HIV infection triggers a diverse antibody response, beginning with early-binding antibodies to envelope glycoproteins (gp120, gp41) and, over time, expanding to antibodies with variable epitope specificities. This immunological diversity has significant implications for diagnostic test design and vaccine development [6]. In particular, antigenic diversity and variability constitute major challenges for early and accurate diagnosis, which is essential for preventing HIV transmission and ensuring timely initiation of treatment [5,7]. 

Fourth-generation HIV screening assays, such as the Abbott Architect and its newer counterpart, the Alinity i HIV Ag/Ab Combo (Abbott Laboratories, Abbott Park, IL, USA), utilize chemiluminescent microparticle immunoassay (CMIA) technology, a modern adaptation of the enzyme-linked immunosorbent assay (ELISA). These assays simultaneously detect antibodies targeting multiple viral proteins—HIV-1 (group M and group O) gp41; gp120; and HIV-2–specific gp36—together with the HIV-1 p24 antigen [8]. This combined detection strategy facilitates early identification during the acute phase of infection, shortening the diagnostic window period and improving overall diagnostic accuracy [7,8].

National and international HIV screening algorithms typically begin with a highly sensitive fourth-generation assay, such as CMIA or ELISA, capable of detecting HIV-1/2 antibodies and the p24 antigen. Rapid immunochromatographic tests are also recommended for point-of-care screening in specific contexts, especially in remote or resource-limited regions [7,9]. Although fourth-generation assays demonstrate high sensitivity, they may still yield false-positive or false-negative results. This issue is particularly critical in low-prevalence populations, where the relative proportion of false results among reactive samples is inherently higher [9,10,11]. Reactive samples are confirmed using a supplemental immunoassay, such as Western blot, line immunoassay, or a rapid immunochromatographic test, and HIV-1 RNA PCR is recommended when results remain indeterminate or acute infection is suspected [7,9,10]. While confirmatory algorithms are essential, they are often time-consuming and resource-intensive, potentially resulting in delayed diagnosis, heightened patient anxiety, and additional strain on health systems [7,9,10]. Incorporating supplementary predictive parameters into screening algorithms may reduce unnecessary confirmatory testing and associated delays, offering a significant advantage in low-prevalence or resource-limited settings [12,13,14,15,16].

One such parameter is the signal-to-cutoff (S/CO) ratio generated by CMIA, where higher values are strongly correlated with confirmed HIV infections and can assist in prioritizing confirmatory testing [12,13]. However, variations in HIV prevalence, target populations, and assay platforms between regions limit the direct transferability of cutoff values [14,15]. The Abbott Architect HIV Ag/Ab Combo assay and the Alinity i HIV Ag/Ab Combo assay (Abbott Laboratories), are among the most widely used CMIA-based platforms worldwide, offering high-throughput automation that reduces turnaround time and operational complexity. While the Architect’s performance and S/CO thresholds have been extensively evaluated [13,14,15,16], no published data have assessed the diagnostic utility of S/CO values for the Alinity system. Nam et al. (2021) demonstrated excellent analytical agreement between the two assays but did not investigate their clinical predictive value [11]. Moreover, no study has established S/CO thresholds for the Architect assay in the Turkish population, limiting the applicability of existing international data to local practice.

This study aimed to establish optimal S/CO thresholds and positive predictive values (PPVs) for the Abbott Architect and Alinity HIV Ag/Ab Combo assays using a large, ten-year dataset. The findings may inform more efficient screening algorithms, and support clinical decision-making in pandemic-affected and low-prevalence settings.

## 2. Materials and Methods

### 2.1. Study Group

**Figure 1:**
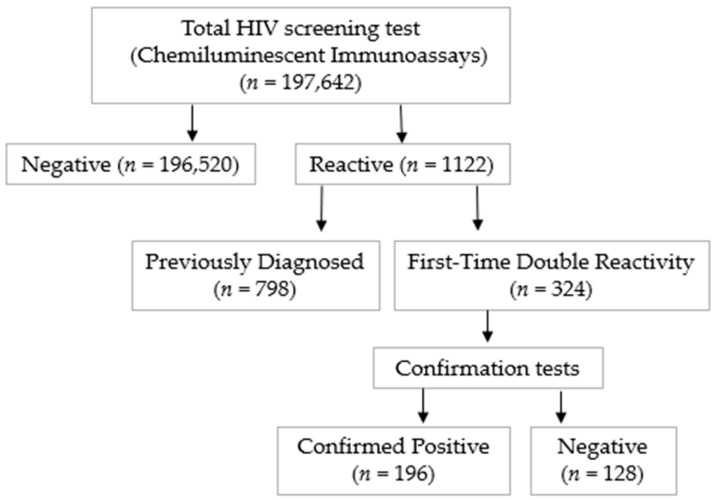
Flowchart of HIV screening and confirmation in this study. Arrows indicate the direction of the screening and confirmation process.

In this retrospective study, we analyzed 197,642 HIV screening test results from individuals processed at a large serology diagnostic laboratory in Bursa Uludag University Hospital, Turkey, between January 2015 and December 2024 (Figure 1).

Each patient was included in the study only once, and no participants were lost during data collection. Demographic details, clinical characteristics, and laboratory test results were retrieved from the routine records maintained by the serology laboratory and the hospital’s online data system, both of which are integral to the HIV diagnostic protocols. Ethical approval was obtained from the Bursa Uludag University Research Ethics Committee (Approval No: 2025/12-26).

### 2.2. Laboratory Analyses

All procedures were performed in accordance with laboratory quality assurance/quality control guidelines and ethical standards at our center. The initial scanning, evaluation, confirmation, and follow-up tests for patients have been conducted in line with the guidelines set by the Turkish Ministry of Health and international standards recommended by global health organizations.

During the study period, initial HIV screening was performed using the Architect HIV Ag/Ab Combo Assay on the ARCHITECT i2000SR instrument (Abbott Laboratories, Abbott Park, IL, USA) from January 2015 to December 2023. Starting in January 2024, screening was transitioned to the next-generation Alinity HIV Ag/Ab Combo assay on the Alinity i system (Abbott Laboratories, Abbott Park, IL, USA), which continued through December 2024. Both fourth-generation CMIA tests simultaneously detect HIV-1 p24 antigen, HIV-1 gp41 antibodies, and HIV-2 gp36 antibodies [8,13,14]. In these assays, paramagnetic microparticles coated with recombinant and synthetic HIV antigens capture target antibodies or antigens from the patient sample. After washing, acridinium-labeled conjugates bind to the captured complexes. Upon addition of trigger solutions, the acridinium label emits light measured in relative light units (RLU), which is proportional to the analyte concentration, with values ≥ 1.00 interpreted as positive.

Although our center is an academic regional hospital with substantial experience in HIV diagnosis and treatment, the official diagnostic algorithm requires that all new HIV 1/2 reactive samples be sent to the National HIV-AIDS Reference Laboratory of the Public Health Institute for verification [10]. Upon obtaining an initial reactive HIV Ab/Ag result, patients are immediately invited to the laboratory; a clinical microbiology specialist provides post-test counseling regarding the preliminary result and provides counseling for the confirmation procedure, including an assessment of high-risk behaviors and contributing factors. A new blood sample is collected, aliquoted, and preserved under recommended conditions for confirmation testing and sent weekly for verification.

### 2.3. Confirmatory Tests

The confirmatory testing algorithm for HIV-reactive samples has evolved, keeping pace with advancements in diagnostic technology and updates to national testing guidelines [7]. As summarized in Table 1, in 2015, confirmation relied exclusively on the HIV-1/2 Line Immunoassay. In 2016, a more comprehensive strategy was introduced, consisting of retesting with the VIDAS HIV Duo Ultra (bioMérieux, Marcy-l’Étoile, France), followed by the HIV 1/2 Line Immunoassay [10]. For samples with negative or indeterminate immunoassay results, HIV RNA testing was also implemented. From 2017 onward, the algorithm was further refined to incorporate the Geenius HIV-1/2 Supplemental Assay (Bio-Rad Laboratories, Redmond, WA, USA) in combination with VIDAS testing. Additionally, when the VIDAS result was positive but the Geenius result was negative or indeterminate, HIV RNA PCR was performed for definitive confirmation.

### 2.4. Statistical Analysis

Normality of continuous variables was evaluated using the Shapiro–Wilk test. Data with normal distribution were presented as mean ± standard deviation, while non-normally distributed data were reported as median (min–max). Depending on data distribution, group comparisons were performed using the Mann–Whitney U test, Kruskal–Wallis test, or one-way ANOVA. Categorical variables were compared using Pearson Chi-Square, Fisher’s exact, or Fisher-Freeman-Halton tests. The Receiver Operating Characteristic (ROC) curves were generated for both the Architect and Alinity HIV Ag/Ab assays to identify optimal S/CO cut-off values. The area under the ROC curve (AUC) was calculated to evaluate diagnostic performance. All statistical analyses were conducted using SPSS version 25.0 (IBM Corp., Armonk, NY, USA), with statistical significance set at *p* < 0.05.

## 3. Results

### 3.1. HIV Testing Trends and Prevalence

Between 2015 and 2024, our institution screened 197,642 patient samples for HIV. Out of these, 1122 tested reactive. Of the reactive cases, 798 were from patients already known to be HIV-positive, while 196 were newly identified infections. This resulted in an overall HIV prevalence of 0.5% (994 out of 197,642) and a new diagnostic rate of 0.1% (196 out of 197,642). This is illustrated in Figure 1, which shows the yearly number of tests and confirmed cases.

**Figure 2:**
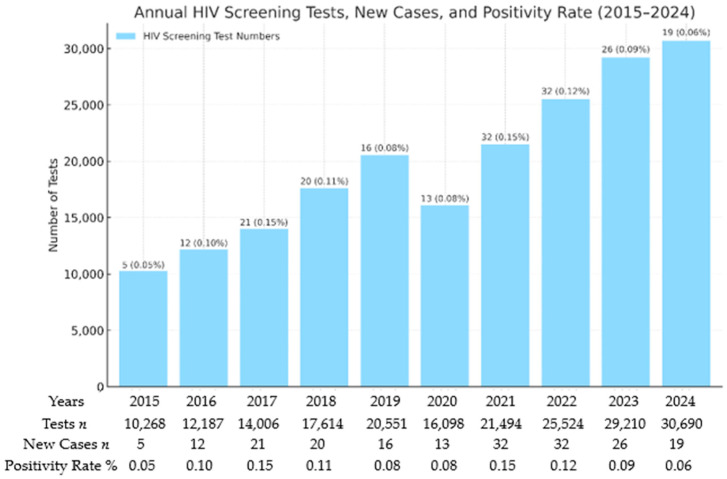
Presents annual trends in HIV screening and new cases between 2015 and 2024. Linear regression analysis revealed a statistically significant rise in the number of HIV tests performed, with an average annual increase of approximately 2200 tests (*p* < 0.001, R^2^ = 0.92). Although there was a brief drop in testing volumes in 2020 due to the COVID-19 pandemic, the overall testing volumes during the pandemic period (2020–2022) were not significantly different from those of non-pandemic years (*p* = 0.667). Additionally, the proportion of new HIV cases relative to the total number of tests varied significantly over time (*p* = 0.019), suggesting variations in new case detection.

A linear regression analysis of HIV test numbers during the study period revealed a statistically significant increase (*p* < 0.001, R^2^ = 0.92), with test counts increasing by about 2200 tests each year on average. Despite a temporary decline in 2020, coinciding with the peak COVID-19 restrictions, the overall difference in testing volumes between pandemic years (2020–2022) and non-pandemic years was not statistically significant (*p* = 0.667) (Figure 2).

**Figure 3:**
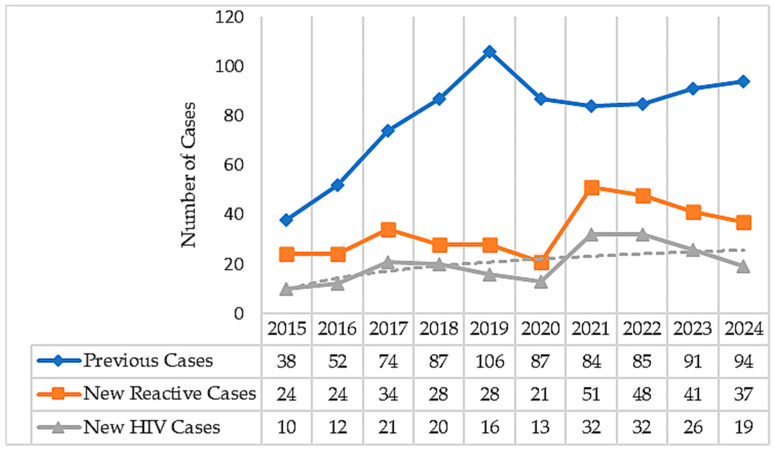
Annual distribution of previously and newly diagnosed HIV cases (2015–2024). Although a numerical decrease was observed during the COVID-19 pandemic period (2020–2022), this drop was not statistically significant for previously diagnosed cases (*p* = 0.909). Overall, testing among individuals with an existing HIV diagnosis showed a significant upward trend (*p* = 0.017, R^2^ = 0.53), suggesting that follow-up and monitoring efforts were largely sustained despite the challenges of the pandemic. The annual number of newly diagnosed HIV cases (*n* = 196) fluctuated over the years. After declining to 13 cases in 2020, the count increased to 32 cases in both 2021 and 2022 (*p* = 0.021). Overall, linear regression analysis revealed a significant upward trend in new HIV diagnoses (*p* = 0.038, R^2^ = 0.43); however, Spearman correlation testing results showed only marginal significance (*p* = 0.062).

The number of newly diagnosed HIV cases (*n* = 196) varied year to year. There was a noticeable increase in 2021 (32 new diagnoses, 0.15%) and again in 2022 (32 new diagnoses, 0.12%), followed by a decrease to 13 cases (0.08%). Subgroup analyses showed that the second year of the pandemic (2021) had the highest rate of new HIV cases (*n* = 32, *p* = 0.021). Overall, linear regression analysis indicated a significant upward trend in new HIV cases (*p* = 0.038, R^2^ = 0.43), although Spearman correlation testing showed only marginal significance (*p* = 0.062). For previously diagnosed individuals (*n* = 798), a consistent increase in retesting was observed over time (*p* = 0.017, R^2^ = 0.53). Although a numerical decrease was noted during the pandemic period (2020–2022), this reduction was not statistically significant (*p* = 0.909) (Figure 3).

### 3.2. Characteristics of Newly Diagnosed HIV Cases

To assess the characteristics of newly diagnosed cases, demographic and immunological data from 196 newly diagnosed patients across seven cities were analyzed (Table 2). Most were Turkish nationals (*n* = 180), and a smaller group of migrants (*n* = 16). The mean age at diagnosis was 38.0 ± 13.3 years, with most patients being male (*n* = 169; 86.2%). The mean age differed significantly between pre-pandemic (38.0 ± 13.3), pandemic (39.8 ± 15.1), and post-pandemic (40.2 ± 11.4) periods (*p* = 0.026).

The most common transmission route was heterosexual contact (53.1%), followed by male-to-male sexual contact (MSM) at 19.4%. MSM transmission dropped to 11.7% during the pandemic but rose to 31.1% post-pandemic (*p* = 0.032). Mean CD4+ T-cell counts at diagnosis declined over time: pre-pandemic [353.0 (10–1210)], pandemic [292.0 (10–2122)], and post-pandemic [210.5 (10–1209)] cells/mm^3^ (*p* = 0.008).

All newly reactive samples were confirmed by a national reference laboratory. The average time from initial reactive result to confirmation was 23.4 ± 10.4 days, with no significant change during the pandemic (*p* = 0.112).

### 3.3. Diagnostic Test Performance and Predictive Value

To assess the diagnostic accuracy of the screening tools used throughout the study period, the diagnostic performance of the Architect and Alinity HIV Ag/Ab Combo tests was evaluated by predictive value and S/CO ratios. Out of 287 reactive samples from the Architect HIV Ag/Ab Combo test, 177 were true positives and 110 false positives, indicating a positive predictive value (PPV) of 61.7% (95% CI: 55.9–67.1%).

**Figure 4:**
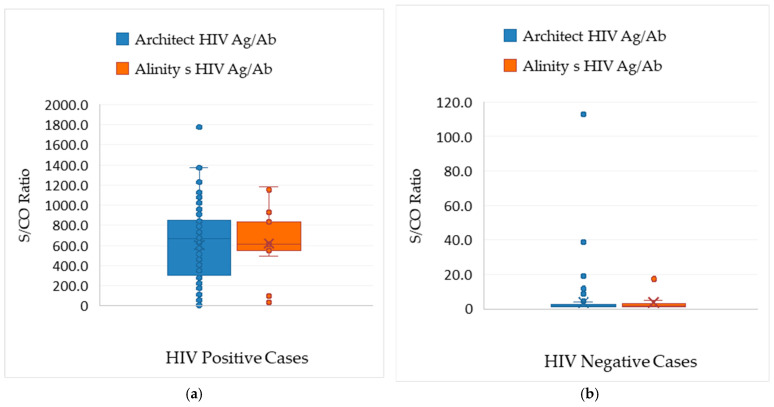
(**a**) The median S/CO ratios in HIV-positive cases did not differ significantly between the Architect and Alinity assays (665.2 vs. 611.0, *p* = 0.887); (**b**) the median S/CO ratios in HIV-negative cases did not differ significantly between the Architect and Alinity assays (1.7 vs. 1.7, *p* = 0.668 for false positives). For both tests, the median S/CO ratios in HIV-positive cases were significantly higher than those in false-positive cases (*p* < 0.001).

The Alinity HIV Ag/Ab Combo test produced 37 reactive samples, 19 true positives, and 18 false positives, with a PPV of 51.4% (95% CI: 35.9–66.5%) (Table 3). Regarding test performance, the Architect assay had significantly higher median S/CO values in confirmed positives than false positives: 665.2 (3.4–1778.6) vs. 1.7 (1.1–113.0). The Alinity assay showed similar results: 611.0 (33.0–1186.0) vs. 1.7 (1.1–19.1) (Figure 4).

**Figure 5:**
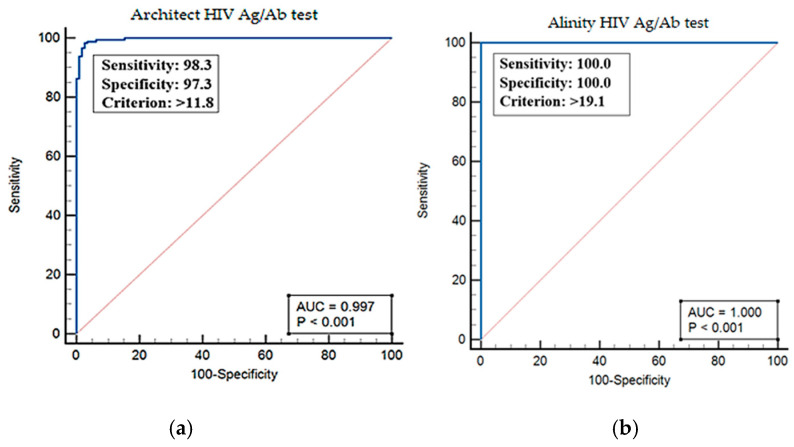
(**a**) Receiver Operating Characteristic (ROC) analysis for the Architect HIV Ag/Ab assay in the detection of HIV. The area under the curve (AUC) was calculated as 0.99, with an optimal cut-off value of >11.8, yielding a sensitivity of 98.3% and a specificity of 97.3%. (**b**) ROC analysis for the Alinity i HIV Ag/Ab assay. The AUC was calculated as 1.00, with a cut-off value of >19.1, achieving 100% sensitivity and 100% specificity for HIV detection.

Receiver Operating Characteristic (ROC) analysis determined optimal cut-off values for both the Architect and Alinity assays. For the Architect assay, a cut-off of 11.8 yielded an AUC of 0.99, with a sensitivity of 98.3% and a specificity of 97.3% (*p* < 0.001). In contrast, the Alinity test showed an AUC of 1.00 at a cut-off of 19.1, achieving 100% sensitivity and specificity, though this finding was based on a smaller sample limited to a single year of testing data (Figure 5). In both tests, PPVs improved with higher S/CO values. For instance, the Architect test reached a PPV of 98.2%, while the Alinity test achieved 100% PPV at higher S/CO thresholds.

## 4. Discussion

This ten-year retrospective study offers an in-depth evaluation of HIV screening trends, diagnostic accuracy, and the predictive utility of S/CO ratios in two commonly used fourth-generation HIV assays, Abbott Architect and Alinity, at a tertiary hospital in Türkiye. The study also highlights the diverse impact of the COVID-19 pandemic on HIV testing, detection, and patient engagement within a low-prevalence context.

### 4.1. Resilience of HIV Testing Systems During the Pandemic

The WHO recommends that HIV diagnostic algorithms be designed to maximize sensitivity and specificity, ensuring prompt confirmation of infection and minimizing false results [7]. However, several international reports have shown significant reductions in HIV testing during the initial phase of the pandemic [17,18], raising concerns about delayed diagnoses and potential increases in transmission. UNAIDS has highlighted that this disruption particularly contributed to delays in diagnosing asymptomatic individuals [18]. In contrast, our findings demonstrate that our center maintained a stable HIV testing infrastructure. Although testing volumes temporarily declined in 2020, corresponding to peak lockdown restrictions, this decline was not statistically significant when compared to pre-pandemic years (*p* = 0.667), demonstrating structural resilience and service continuity of our services.

This finding is consistent with WHO recommendations that emphasize sustaining essential diagnostic services during public health crises [7]. The ability of our institution to continue HIV screening without interruption demonstrates a robust, well-integrated system supported by laboratory automation, skilled staff, and effective coordination among clinical, laboratory, and confirmatory departments.

### 4.2. Care Engagement Among Previously Diagnosed Individuals

The significant increase in the number of individuals with a prior HIV diagnosis returning for HIV screening (*p* = 0.017, R^2^ = 0.53) likely reflects global efforts to strengthen care retention and expand access to antiretroviral therapy (ART) [5,17]. Notably, this trend persisted throughout the pandemic, showing minor variations. These results are consistent with the 2021 UNAIDS Global AIDS Update, which reported that despite initial disruptions, most health systems maintained continuity of care for people already diagnosed with HIV [18]. This underscores the resilience of follow-up systems and ART programs, even as testing and diagnosis among asymptomatic or undiagnosed individuals may have been affected.

### 4.3. Fluctuations in New HIV Diagnoses and Late Presentations

In contrast to previous trends in HIV diagnoses, the number of newly diagnosed HIV cases fluctuated during the pandemic. After a drop in 2020 (*n* = 13), cases peaked in 2021 and 2022 (*n* = 32 each; *p* = 0.021). In 2022, the rate of new HIV cases relative to the total number of tests was 0.15% (32/21,494), significantly higher than in other years (*p* = 0.019). This increase likely reflects a shift toward testing higher-risk or symptomatic individuals when routine screening services were limited—consistent with international reports of testing disruptions and delayed diagnoses [4,17,18]. U.S. national surveillance data confirmed this pattern, showing a 17% decline in HIV testing between 2019 and 2020, particularly among individuals who did not actively seek care [19]. Interestingly, while overall testing volume declined, the proportion of positive results increased, suggesting a higher pre-test probability during the pandemic period [4,20].

### 4.4. Shifts in Transmission Patterns

Previous epidemiological data have highlighted shifts in the demographic profile of individuals diagnosed with HIV in Türkiye. For example, a multicenter retrospective study by Erdinc et al., covering the years 2011–2016 found that the majority of patients were male (male-to-female ratio 5:1), the highest prevalence was observed in the 25–34 age group, and the proportion of cases associated with MSM transmission increased significantly over the study period [21]. In our cohort, the mean age at HIV diagnosis was 34.7 ± 11.8 years in the pre-pandemic period, consistent with the findings of Erdinc et al. However, a significant increase was observed during the pandemic and post-pandemic periods, with mean ages rising to 39.8 ± 15.1 and 40.2 ± 11.4 years, respectively (*p* = 0.026). This upward trend may reflect diagnostic delays as well as increased vulnerability among older populations.

Simultaneously, a significant drop was observed in CD4+ T-cell counts at diagnosis, from 353/mm^3^ before the pandemic to 210/mm^3^ afterward (*p* = 0.008). Since CD4 < 350/mm^3^ indicates a late-stage diagnosis, this points to a clinically relevant delay in detection. Late diagnosis is associated with increased morbidity, mortality, and healthcare burden, and it complicates timely antiretroviral therapy (ART) initiation and transmission control efforts [22].

Pandemic-era demographic data also revealed fluctuations in transmission routes. The proportion of MSM-related transmission decreased during the early pandemic period but increased again after the pandemic (*p* = 0.032). This trend may be associated with differences in care access and interruptions in outreach services for specific populations. Rao et al. reported that MSM communities in countries like Türkiye experienced significant disruptions in testing, pre-exposure prophylaxis (PrEP), and counseling during the pandemic, underscoring systemic vulnerabilities [20]. Similar concerns were highlighted in the UNAIDS Global AIDS Update, which documented service disruptions among key populations, including MSM, during the COVID-19 crisis [18].

### 4.5. S/CO Ratio as a Diagnostic Tool

One of the central objectives of this study was to evaluate the predictive value of S/CO ratios. Our ROC analysis confirmed a strong correlation between elevated S/CO values and confirmed HIV positivity for both assays. The Architect assay demonstrated an optimal S/CO cut-off of ≥11.8, achieving 98.3% sensitivity and 97.3% specificity, comparable to thresholds reported in prior studies as presented in Table 4 [13,14,15,16]. While very high cut-offs (e.g., >100) may achieve near-perfect PPVs, they risk missing cases in early seroconversion [14]. Consistently, Guo et al. (2023) demonstrated the predictive value of ≥14.09 S/CO ratios (93.8% sensitivity, 93.1% specificity, and 97.9% PPV) on a different platform, further supporting their utility in HIV diagnosis [12]. The selected threshold in our study provides a balance between diagnostic accuracy and early detection. For the Alinity platform, an S/CO threshold of ≥19.1 yielded 100% sensitivity and specificity. To our knowledge, this is one of the first ROC-based thresholds reported for Alinity in the literature, as previous studies only addressed analytical agreement without clinical predictive assessment [13]. Moreover, no study has previously established S/CO thresholds for the Architect assay in the Turkish population, limiting the applicability of international data to local practice. According to the diagnostic algorithm, all reactive results must undergo confirmatory testing. However, the S/CO thresholds (≥11.8 for Architect and ≥19.1 for Alinity) may allow early referral of patients with high results for treatment initiation without waiting for confirmation. Given that the confirmation process takes an average of 23.4 ± 10.4 days, this approach could help minimize delays and prevent potential complications.

These findings support the incorporation of S/CO-guided triage as part of routine HIV screening, particularly in high-volume or resource-limited settings [12,13,14,15,16]. Elevated S/CO values can help predict true positives and reduce unnecessary confirmatory testing, which can minimize patient anxiety and accelerate diagnosis. This approach is particularly valuable in systems where confirmatory testing is centralized or subject to delays or, such as in our center, where reactive samples are sent to a national reference laboratory [7,10].

### 4.6. Confirmation Rates and Platform Transition

Across the ten-year study period, the confirmation rates for reactive samples remained constant across pre-pandemic, pandemic, and post-pandemic periods (*p* = 0.415), suggesting consistent diagnostic reliability. A minor decline in 2024 coincided with the transition from the Architect to the Alinity platform, highlighting the importance of monitoring performance during platform changes [13].

### 4.7. Strengths and Limitations

Key strengths of this study include the large sample size, decade-long duration, and the combined use of epidemiological and laboratory data, which enabled robust trend analysis and ROC modeling. Nevertheless, several limitations should be noted. First, the retrospective, single-center design and the relatively small number of confirmed positive cases for the Alinity assay restrict the generalizability of its calculated S/CO threshold, underscoring the need for validation in larger cohorts. Second, while S/CO values provide valuable support for triage, confirmatory testing remains indispensable, particularly for results within low or borderline ranges [12,14]. Finally, changes in confirmatory testing algorithms over the 10-year period may have influenced overall confirmation rates.

## 5. Conclusions

Our findings highlight the diagnostic resilience of HIV testing programs even during public health crises and demonstrate the value of using S/CO ratios to improve testing efficiency. Both the Architect and Alinity assays demonstrated high predictive accuracy at validated thresholds. Integration of S/CO-based triage into diagnostic algorithms may streamline clinical workflows, reduce unnecessary confirmatory testing, and enhance timely linkage to care, particularly in low-prevalence or resource-limited settings. As global health systems brace for future challenges, the development of adaptable, evidence-based, and locally adapted HIV testing strategies will remain essential.

## Figures and Tables

**Table 1:** Confirmatory tests by years.

Year	Confirmatory Tests Used
2015	HIV 1/2 Line Immunoassay
2016	VIDAS HIV Duo Ultra + HIV1/2 Line Immunoassay + HIV RNA
2017–2024	VIDAS HIV Duo Ultra + Geenius HIV-1/2 Supplemental Assay + HIV RNA

**Table 2:** The characteristics of new HIV patients and their changes in the pre-pandemic, pandemic, and post-pandemic periods.

Variable		Total *n* = 196, (%)	Pre-Pandemic 2015–2019 *n* = 74	Pandemic 2020–2022 *n* = 77	Post-Pandemic 2023–2024 *n* = 45	*p*
**Gender**	Male	169 (86.2)	66 (89.2)	65 (84.4)	38 (84.4)	0.644
	Female	27 (13.8)	8 (10.8)	12 (15.6)	7 (15.6)	
**Age**	Mean (±)	38.0 ± 13.3	34.7 ± 11.8	39.8 ± 15.1	40.2 ± 11.4	0.026
**Ethnicity**	Turkiye	180 (91.8)	68 (91.9)	72 (93.5)	40 (88.9)	0.668
	Others	16 (8.2)	6 (8.1)	5 (6.5)	5 (11.1)	
**Married**	Yes	98 (50)	42 (56.8)	40 (50.9)	16 (35.5)	0.073
	No	98 (50)	32 (43.2)	37 (49.1)	29 (65.0)	
**Education**	Middle/high school	116 (69)	42 (59.2)	50 (78.1)	24 (72.7)	0.052
	University	52 (31)	29 (40.8)	14 (21.9)	9 (27.3)	
**Exposure**	Heterosexual contact	104 (53.1)	44 (59.5%)	39 (50.6)	21 (46.7)	0.344
	Homosexual contact (MSM)	38 (19.4)	15 (20.3)	9 (11.7)	14 (31.1)	0.032
	IVDU	15 (7.7)	4 (5.4)	8 (10.4)	3 (6.7)	0.495
	Tattoo	15 (7.7)	3 (4.1)	10 (13.0)	2 (4.4)	0.078
	Other	5 (2.6)	2 (2.7)	2 (2.6)	1 (2.2)	0.990
	Unknown	44 (22.4)	13 (17.6)	22 (28.6)	9 (20.0)	0.243
**CD4 count** (cells/mm^3^)	Median (min–max)	315.0 (10–2122)	353.0 (10–1210)	292.0 (10–2122)	210.5 (10–1209)	0.008
**Architect HIV Ag/Ab** S/CO	Median (min–max)	665.2 (3.4–1778.6)	563.4 (6.5–1778.0)	682.7 (3.4–1176.2)	745.1 (30.2–1230.2)	0.136
**Time taken for** **Confirmation** ^c^ **(day)**	Mean (±)	23.4 (10.4)	24.2 (9.8)	21.6 (9.9)	22.5 (11.2)	0.112
**Confirmation rate**	**Architect ^a^** *n*, %	177/287 (61.7)	74/126 (58.7)	77/120 (64.2)	26/41 (63.4)	0.614
	**Alinity ^b^ ***n*, %	-	-	-	1937 (51.3)	-

IVDU: Intravenous drug use. Other: Mother-to-child, injector needle stick, blood transfusion. Data are expressed as mean ± st deviation, median (minimum-maximum), and *n*%. ^a^ It covers the years 2015–2023. **^b^** It covers the year 2024. ^c^ Calculated from the date of the initial reactive result.

**Table 3:** Comparison of S/CO values for the Architect and Alinity tests.

	Architect HIV Ag/Ab ^a^	Alinity i HIV Ag/Ab ^b^	*p*
HIV-positive	*n* = 177	*n* = 19	0.887
Median (min-max)	665.2 (3.4–1778.6)	611.0 (33.0–1186.0)	
Mean (std dev.)	604.1 ± 351.0	618.4 ± 317.2	
False positive	*n* = 110	*n* = 18	0.668
Median (min-max)	1.7 (1.1–113.0)	1.7 (1.1–19.1)	
Mean (std dev.)	3.8 ± 11.3	3.8 ± 5.4	

Data are expressed as mean ± st deviation and median (minimum-maximum). ^a^ It covers the years 2015–2023, ^b^ It covers the year 2024.

**Table 4:** Performance of S/CO ratios in the Architect and Alinity HIV Ag/Ab Combo assays in the previous and this study.

	Year	Test	Threshold S/CO	Sensitivity %	Specificity %	PPV %	Notes
Jensen et al. [14]	2015	Architect	>151.2	67.4	-	100.0	High threshold suggested for low prevalence
Hodgson et al. [13]	2020	Architect	>100.0	100.0	-	100.0	High threshold ensures all reactive samples are true positives
Alonso et al. [15]	2018	Architect	>2.05	100.0	100.0	95.8	Low threshold suggested for identifying newly infected cases
Whitney et al. [16]	2022	Architect	>3.78	96.3	93.9	94.1	Associated with clinical decision support systems
This study	2025	Architect	>11.8	98.3	97.3	98.2	Large sample size, including pandemic data
This study	2025	Alinity	>19.1	100.0	100.0	100.0	Initial ROC value for Alinity

PPV: Positive Predictive Value, S/CO: Signal-to-Cutoff.

## Data Availability

The datasets used and/or analyzed during the current study are available from the corresponding author on reasonable request.

## Abbreviations

The following abbreviations are used in this manuscript:

AIDS	Acquired Immunodeficiency Syndrome
ART	Antiretroviral Therapy
AUC	Area under the Receiver Operating Characteristic curve
CD4	Cluster of Differentiation 4 (T-helper cell surface marker)
CMIA	Chemiluminescent Microparticle Immunoassay
ELISA	Enzyme-Linked Immunosorbent Assay
HIV	Human Immunodeficiency Virus
LIA	Line Immunoassay
MSM	Men who have Sex with Men
PPV	Positive Predictive Value
PrEP	Pre-exposure prophylaxis
ROC	Receiver Operating Characteristic
S/CO	Signal-cutoff
UNAIDS	Joint United Nations Program on HIV/AIDS
WHO	World Health Organization
